# Effect of storage on the physicochemical, rheological, antioxidant activity, and sensory properties of soy whey‐fortified pineapple juice beverages

**DOI:** 10.1002/fsn3.4219

**Published:** 2024-05-27

**Authors:** Jahangir A. Rather, Hilal Ahmad Punoo, Najmeenah Akhter, Sabeera Muzzaffar, Firdous Ahmad Khanday, Gulden Goksen

**Affiliations:** ^1^ Department of Food Science and Technology University of Kashmir Srinagar India; ^2^ Department of Food Technology Islamic University of Science and Technology Awantipora, Pulwama India; ^3^ Department of Food Technology, Vocational School of Technical Sciences at Mersin Tarsus Organized Industrial Zone Tarsus University Mersin Turkey

**Keywords:** antioxidant activity, beverages, shelf life, soy whey, storage studies

## Abstract

Incorporating soy whey into pineapple juice can enhance nutritional and nutraceutical potential. The present study was conducted to develop soy whey‐fortified pineapple juice beverage and impact of ambient storage conditions on physicochemical, antioxidant, rheological, and sensory properties of functional beverage. Increasing the storage period decreased pH from 4.1 to 3.7 for control and 4.0 to 3.8 for soy whey‐treated samples. TSS increased from 8.3 to 10.6 on 0th day for control and soy whey‐treated beverage samples while on the 30th day, the TSS ranged from 8.9 to 11.1°B. Up to 30% soy whey incorporation, the DPPH, reducing power, and ABTS of beverages increased from 5.58%–57.01%, 56.35%–56.90%, and 4.84%–47.01%, respectively. The flow index (*n*) of the beverage formulations ranged between 0.4758 and 0.6521, and the yield stress between 0.018 and 0.025 Pa, hence showing Herschel–Bulkley character. With the increase in concentration and storage period, both *G′* and *G*″ values decreased considerably (*p* < .05). The standard plate and yeast and mold count decreased considerably with an increase in soy whey and increased with an increase in storage. The sensory score of the beverages up to 30% soy whey exhibited best sensory score results compared to control and samples with 30% above soy whey content.

## Graphical abstract

Phytonutrient‐rich beverages are gaining importance day by day owing to the health‐conscious behavior of consumers. The present study aimed to develop a nutraceutical‐rich pineapple beverage by incorporating soy whey as a waste byproduct of tofu preparation.

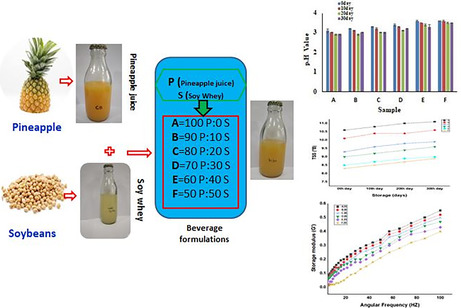

## 1 INTRODUCTION

Fruit juices are rich sources of vitamins, minerals, and bioactive components (Arumugam et al., 2021). Fresh fruit juice consumption is increasing globally due to consumer perception of freshness, high vitamin content, low‐calorie value, and the ability to decrease the risk of many diseases (Rather, Yousuf, et al., 2022). They could prevent the occurrence of numerous diseases, such as heart disease, cancer, and diabetes (Ruxton & Myers, 2021). Pineapple is rich in vitamin C, folate, thiamin, riboflavin, proteins, fibers, and other minerals like copper, potassium, magnesium, and iron (Rather et al., 2023). Moreover, pineapple has a high bioactive component content, which boosts its antioxidant capacity (Wu et al., 2021). The popularity of pineapple has grown in recent years due to its appealing flavor, high nutritional value, and antioxidant content (Difonzo et al., 2019).

Due to the presence of daidzein, genistein, glycitin, and epigenistin components, food products made from soybeans offer a diversity of biological actions like antioxidant, anticarcinogenic, antihypertensive, antidiabetic, and antiobesity capacities (Azi et al., 2021). Additionally, it has been demonstrated that soy protein shields the anthocyanin components from degradation as they transit through the upper digestive tract and are subsequently delivered colonially (Grace et al., 2021; Troup, 2018). The soy whey contains a substantial amount of nutrients and numerous bioactive components such as phenolics, polypeptides, and phospholipids (Chourasia et al., 2022). These vital ingredients give food products made from soybeans a high level of health‐related properties (Azi et al., 2021). The soybean contains significant amounts of soy isoflavones, which have been shown to have a major effect on radical scavenging action, cancer risk reduction, and the treatment of osteoporosis and menopausal symptoms in women. It has been observed that the intestinal epithelium absorbs the isoflavone aglycones immediately, without needing to have time to hydrolyze the glycoside molecule. Comparing them to isoflavone glycosides, it is known that they exhibit more biological activity (Dai et al., 2021).

Drinking beverages provides instant energy to the body and shows refreshing effect (Arora et al., 2020). Development of pineapple beverages incorporated with soy ingredients is a novel approach for the development of nutraceutical‐rich pineapple–soy whey beverage. The creation of these drinks is urgently needed as the popularity of nutrient‐dense, healthful, and environmentally friendly diets grows. Drinks high in protein and phytochemicals with several transformative qualities are becoming more and more popular (Sun et al., 2022). The pineapple beverages have been developed from pineapple and cheese whey (Islam et al., 2021); however, the pineapple–soy whey beverage has not yet been developed and evaluated for its physiochemical and sensory properties. Additionally, the beverages are being stored under refrigerated storage conditions for quality retention and shelf life enhancement. The present study was conducted to evaluate the impact of ambient (25 ± 2.0°C) storage conditions on physicochemical, nutraceutical, rheological, antioxidant, and sensory properties of soy whey‐fortified pineapple juice beverages.

## 2 MATERIALS AND METHODS

The pineapple fruit and the soybeans were bought from Hazratbal Srinagar's local market (190006). Before being processed further, the pineapple fruits were cleaned and washed, the soybean seeds were cleansed and any extraneous materials were physically removed.

### 2.1 Development of soy whey (SW) and pineapple juice (PJ)

The soy whey (SW) was created using a methodology akin to that of Kumar and Dhingra (2014). In order to disguise the beany flavor of the soybeans, the cleaned soybean seeds were briefly cooked in hot water at 1:6 ratios for 30–35 min. After soaking in the same water for 12 h, the soybeans were blended to form soy milk. Then, 2% citric acid was incorporated into the soymilk, the tofu was separated, and the whey was gathered.

The pineapples were cleaned with water, peeled, cored, and cut into slices. After that, the slices were utilized to extract juice using a juice extractor, analogous to Tanwar et al. (2022). The juice was then clarified using a muslin cloth and kept at refrigerated storage (7 ± 1°C) till used.

### 2.2 Proximate composition of soy whey and pineapple juice

The moisture, protein, fat, ash, pH, and TSS of the pineapple juice and soy whey were determined similar to the method of Punoo et al. (2023).

### 2.3 Development of soy whey‐fortified pineapple juice beverage

Six combinations of beverages were developed from pineapple juice and soy whey with 5% sugar in each formulation. The beverage formulations were coded as A, B, C, D, E, and F, where A = 0 mL SW, 100 mL PJ, B = 10 mL SW, 90 mL PJ, C = 20 mL SW, 80 mL PJ, D = 30 mL SW, 70 mL PJ, E = 40 mL SW, 60 mL PJ, and F = 50 mL SW, 50 mL PJ. The developed beverages were heated at 90°C for 15 min and were kept at refrigerated conditions (25 ± 2.0°C). The beverages were then analyzed at 0, 10, 20, and 30 days for the various parameters.

### 2.4 Storage studies

The soy whey‐fortified pineapple juice beverages were kept at ambient storage conditions (25 ± 2°C) in order to evaluate the impact of ambient storage conditions on the quality and shelf life of beverages and were evaluated for various parameters during storage of 30 days.

#### 2.4.1 pH and total soluble solids (TSS)

The pH meter (LABMAN LMPH‐12, India) was used to measure the pH of beverage, following the methodology of Rather, Majid, et al. (2022), Rather, Makroo, et al. (2022), and Rather, Yousuf, et al. (2022). A handheld refractometer (ATAGO‐0258999, Japan) was used to measure the TSS of beverage samples.

#### 2.4.2 Instrumental color

Using the Flex EZ Model No. 45/0 Hunter color lab, the sample color was ascertained. The L* (lightness), a* (redness), and b* (yellowness) were measured using a methodology akin to that of Rather, Majid, et al. (2022), Rather, Makroo, et al. (2022), and Rather, Yousuf, et al. (2022).

#### 2.4.3 Antioxidant properties

The antioxidant activity of the beverage formulations was done by evaluating their DPPH, reducing power, and ABTS (%) activity.

##### α, α‐diphenyl‐β‐picrylhydrazyl (DPPH) activity

Three milliliters of each beverage formulation was combined with 1 mL of an ethanol solution having 0.1 mM DPPH. After giving the mixture a vigorous shake and letting it stand in the dark for 30 min, the absorbance at 517 nm was taken using a ultraviolet–visible spectrophotometer (Hitachi, U2900). The DPPH activity in percentage was computed as:

(1) $$\text{DPPH}\;\left(\%\right)=\frac{\text{absorbance}\;\text{of}\;\text{sample}-\text{absorbence}\;\text{of}\;\text{control}}{\text{absorbance}\;\text{of}\;\text{control}}\times 100$$

##### Reducing power (RP)

RP of samples was determined similar to the method of Baba, Rashid, et al. (2016) with few alterations. Briefly, 500 μL of the sample was dissolved in 0.2 M phosphate buffer (2.5 mL) solution, and then 2.5 mL of 10% potassium ferricyanide was added to it. The mixture was stored for 20 min (50°C) followed by adding 25 mL of 10% trichloroacetic acid and then centrifuged at 1107 *g* (10 min). Then, supernatant (2.5 mL) was taken and 2.5 mL of purified water and 0.1% FeCl_3_ solution (0.5 mL) were added to the mixture. The absorbance was taken at 700 nm and reduction (%) was calculated by following formulae:

(2) $$\text{Reduction}\;\left(\%\right)=A\left(\text{test}\right)/A\left(\text{blank}\right)\times 100$$

where A (test) is the sample absorbance and A (blank) is the control absorbance.

##### 2,2′‐azino‐bis 3‐ethylbenzothiazoline‐6‐sulfonate (ABTS)

ABTS activity was obtained similar to that of Baskar et al. (2011) with minor alterations. The ABTS (7 mM) and (NH_4_)_2_S_2_O_8_ (2.45 mM) were mixed and kept for 12 h in the dark. The methanol was added to the developed ABTS solution in 1:60 ratio, and 150 μL of each beverage sample was mixed with 2850 μL of ABTS solution. The mixture was kept in dark for 6 min and absorbance was taken at 723 nm. The inhibition (%) was determined by the following formula:

(3) $$\text{Inhibition}\;\left(\%\right)=\left[\mathrm{Ac}-\mathrm{As}/\mathrm{Ac}\right]\times 100$$

where Ac is the control absorbance and As is the sample absorbance.

#### 2.4.4 Microbiological analysis

The total plate count (Log CFU/mL) and yeast and mold count (Log CFU/mL) of the beverage samples were determined similar to the method of Ahmed et al. (2023).

#### 2.4.5 Rheological properties

The rheology of a beverage directly affects its consumer acceptance, making rheological qualities crucial analytical factors for the examination of beverage samples. The flow behavior and frequency sweep were as follows:

##### Flow behavior (FB)

The FB of beverage formulations was determined similar to that of Rather, Majid, et al. (2022), Rather, Makroo, et al. (2022), and Rather, Yousuf, et al. (2022) at 25°C within a frequency range of 0.1–100 rad/s.

##### Frequency sweep (FS)

For the majority of food products, the dynamic rheology is a crucial component in measuring food behavior (Mitra et al., 2022). The frequency tests of beverage formulations were determined by dynamic rheological measurements alike to Rather, Majid, et al. (2022) with little variation. Briefly, 2 mL of beverage formulations was used, the amplitude test was first performed from 0.1% to 20% strain with 0.1 Hz frequency for evaluation of LVE region. From amplitude sweep, 1% strain was selected for FS tests. The tests were accompanied at 25°C from 0.1–100 rad/s to determine storage (*G*′) modulus and loss (*G*″) modulus values.

#### 2.4.6 Sensory evaluation

The sensory evaluation of the beverage samples was done by the senior academic members and research scholars of Department of Food Science and Technology, University of Kashmir. Each 25 participants received 50 mL of each sample for the sensory assessment. During the sensory evaluation, the beverages were assessed for color, flavor, taste, consistency, and overall acceptability using 9‐point hedonic scale similar to Punoo et al. (2023).

#### 2.4.7 Statistical analysis

The results are communicated as the mean of the replicates, and each test was run three times. The ANOVA was conducted using the SPSS statistics program (v.16, SPSS Inc., Chicago, IL, USA). A posthoc Duncan's test was used to determine the significance of the data points at a significance level of 5% (*p* < .05).

## 3 RESULTS AND DISCUSSIONS

### 3.1 Proximate composition of soy whey and pineapple juice

The proximate composition of the freshly extracted pineapple juice and soy whey is shown in Table 1. The moisture content of the developed pineapple juice was 84.32%, Ogunmefun et al. (2018) reported a moisture content of 89.55% in freshly extracted pineapple juice. The protein, fat, and ash content of the pineapple juice were 0.43%, 0.0%, and 0.23%, respectively. Similarly, Ogunmefun et al. (2018) reported protein, fat, and ash content of 0.52%, 0.00%, and 0.19%, respectively, for freshly extracted pineapple juice. The TSS value of the pineapple juice was 11.4, the results are in line with Oyeleke et al. (2013), who reported a TSS of 10.62°B for pineapple juice. The pH of the pineapple juice was 3.71, Oyeleke et al. (2013) documented pH values of 5.20 and 3.80 for pineapple and watermelon juice, respectively. Additionally, Jori et al. (2013) reported pH of 4.10 for the pineapple pulp with a TSS of 15°B. This variation in pH may be attributed to the variation in the ripening of pineapple fruit as reported by Solomon et al. (2016).

**TABLE 1:** Proximate composition of soy whey and pineapple juice.

Constituents (%)	Soy whey	Pineapple juice
Moisture	88.59 ± 0.01	84.32 ± 0.12
Protein	0.45 ± 0.05	0.43 ± 0.04
Fat	0.32 ± 0.13	0.00 ± 0.00
Ash	0.27 ± 0.05	0.23 ± 0.02
TSS	4.12 ± 0.02	11.4 ± 0.01
pH	6.14 ± 0.02	3.71 ± 0.05

*Note*: The data are presented as mean ± standard deviation (*n* = 3).

The pH of the soy whey was 6.14 lower than the pH reported by Kumar and Dhingra (2014), which may be owing to the type and percentage of the acid used for the extraction of the soy whey. Additionally, Chua et al. (2017) reported pH value of 3.88 for pretreated soy whey. The variation in the pH of the soy whey may be owing to the type and concentration of acid used in pretreatment during the development of soy whey (Punoo et al., 2023). The TSS, protein, ash, and fat content of the extracted whey were 4.12%, 0.45%, 0.27%, and 0.32%, respectively. Kumar and Dhingra (2014) reported TSS and protein content of 1.77% and 3.0%, respectively. Additionally, Chua et al. (2017) reported a protein content of 1.33% of the soy whey produced during the tofu preparation.

### 3.2 Storage studies of beverages

#### 3.2.1 pH analysis

**FIGURE 1:**
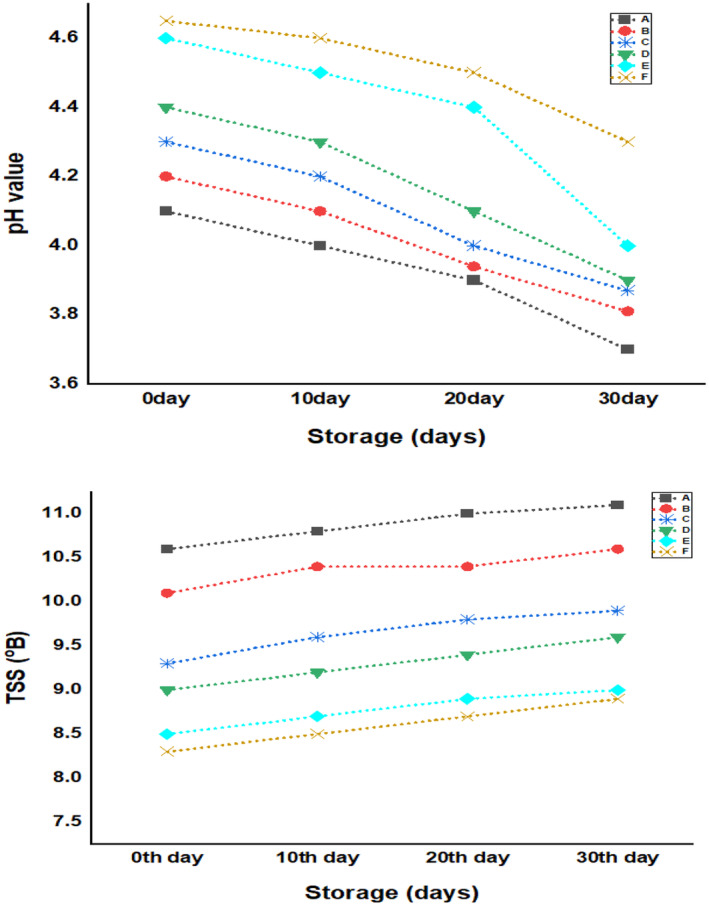
pH and TSS values of beverage samples, A (0‐mL soy whey, 100‐mL pineapple juice), B (10‐mL soy whey, 90‐mL pineapple juice), C (20‐mL soy whey, 80‐mL pineapple juice), D (30‐mL soy whey, 70‐mL pineapple juice), E (40‐mL soy whey, 60‐mL pineapple juice), and F (50‐mL soy whey, 50‐mL pineapple juice).

The pH of SW‐treated pineapple juice beverages is shown in Figure 1. With an increase in the whey percentage of beverages, the pH increased significantly (*p* < .05) from 4.2 to 4.6. Similar increase in the pH of orange beverage treated with soy whey was reported by Punoo et al. (2023). The rise in pH was due to the higher pH of soy whey than pineapple juice as stated by Punoo et al. (2023) and Ribeiro et al. (2014). Yadav et al. (2014) stated pH values of 4.0–4.8 for SW and soy‐milk‐treated fruit juice beverages and is owing to rise in their TSS which potentates the release of bounded nutrients. Similarly, Rohit et al. (2019) stated pH of 4.37–4.97 for whey‐treated kiwi juice beverages. During storage, the pH values of developed beverage formulations decreased significantly (*p* < .05) from 4.1 to 3.7 for control and 4.6 to 4.1 for soy whey‐treated samples. Similar decrease in pH was also stated by Ahmed et al. (2023) in milk whey‐based fruit juice beverages during 25‐day storage. Ahmed et al. (2023) stated that the decrease in the pH may be attributed to the generation of amino and organic acids from proteins and other components of beverages. This decrease in pH is a beneficial attribute for enhancing the shelf life of beverages (Mishra & Sangma, 2017).

#### 3.2.2 Total soluble solids (TSS)

The TSS of the developed SW‐treated pineapple beverage formulations is shown in Figure 1. The TSS of pineapple beverage formulations decreased from 10.6 to 8.3 with an increment in the percentage of SW. Similar decrease in TSS was also described by Punoo et al. (2023) in orange‐based beverages treated with SW, owing to lower TSS of soy whey compared to the orange juice. Yadav et al. (2016) stated TSS ranging between 11°B and 16°B for SW and soy‐milk‐treated fruit‐based beverages. The TSS of whey‐treated kiwi juice beverage ranges between 8.88°B and 16.53°B, as stated by Rohit et al. (2019). Additionally, TSS of soy‐based papaya beverage formulations was 9.17 and 10.0°B treated with 30% and 35% mango juice, respectively (Ribeiro et al., 2014). With an escalation in the storage days, the TSS of the developed beverage formulations increased slightly. This increase in TSS correlates with pH results, as with storage pH decreases and hence there is hydrolysis of polymeric substances in beverages (Yadav et al., 2013). Dhiman et al. (2017) reported that the increase in TSS is attributed to the solubilization of solids and conversion of polysaccharides to simple sugars. Rehman et al. (2014) also stated an increase in the TSS of fruit juice concentrate during 30‐day storage period.

#### 3.2.3 Instrumental color

The color values (L*, a*, and b*) of the soy‐whey‐treated pineapple beverages are shown in Table 2. Color is an essential quality characteristic for the consumer acceptability of the beverage products, alterations in the color values decrease their consumer acceptability (Porto et al., 2017). With an escalation in the percentage of SW from 10% to 50%, the L* value increased considerably (*p* < .05) from 14.65 to 25.24. Baba, Din, et al. (2016) and Baba, Rashid, et al. (2016) also stated a rise in L* value of paneer and cheese whey‐fortified pineapple beverages with increase in percentage of whey. Vieira et al. (2020) also stated greater L* values of orange juice beverages treated with goat milk whey than the control beverage samples. This escalation in the L* value may be ascribed to the greater L* of the SW than the juice and maybe due to the dilution effect, hence increase in SW percentage, L* value increased considerably (Punoo et al., 2023). Additionally, García‐Pérez et al. (2005) stated an increase in L* of the yogurt with fiber incorporation, which is attributed to higher water absorption of fiber. Hence, the greater the water percentage in the beverages, the higher will be the L* of the beverage formulations.

**TABLE 2:** Instrumental color analysis of beverage samples during storage.

Parameter	Sample	0th day	10th day	20th day	30th day
L* value	A	14.65 ± 0.20^Fa^	14.34 ± 0.05^Fab^	14.12 ± 0.03^Fac^	13.54 ± 0.12^Fad^
	B	17.37 ± 0.05^Ea^	17.23 ± 0.04^Eab^	16.48 ± 0.02^Ec^	14.12 ± 0.07^Ed^
	C	20.68 ± 0.03^Da^	20.51 ± 0.11^Da^	18.43 ± 0.05^Db^	17.34 ± 0.02^Dc^
	D	22.24 ± 0.02^Ca^	22.20 ± 0.05^Ca^	21.84 ± 0.10^Cb^	20.32 ± 0.08^Cc^
	E	23.33 ± 0.04^Ba^	23.20 ± 0.06^Bab^	22.12 ± 0.02^Bc^	20.46 ± 0.11^Bd^
	F	25.24 ± 0.03^Aa^	25.16 ± 0.04^Aa^	23.41 ± 0.07^Ab^	22.12 ± 0.02^Ac^
a* value	A	−1.68 ± 0.03^Ad^	−1.52 ± 0.05^Ac^	−1.11 ± 0.02^Ab^	−1.02 ± 0.04^Aa^
	B	−1.77 ± 0.04^Cd^	−1.74 ± 0.01^Bc^	−1.66 ± 0.06^Bb^	−1.52 ± 0.06^Ba^
	C	−1.83 ± 0.02^Ed^	−1.80 ± 0.03^Fc^	−1.76 ± 0.10^Fb^	−1.70 ± 0.08^Fa^
	D	−1.75 ± 0.02^Bd^	−1.71 ± 0.05^Cc^	−1.67 ± 0.02^Cb^	−1.54 ± 0.11^Ca^
	E	−1.82 ± 0.04^Dd^	−1.80 ± 0.01^Ec^	−1.76 ± 0.04^Eb^	−1.72 ± 0.02^Ea^
	F	−1.89 ± 0.03^Fd^	−1.85 ± 0.06^Dc^	−1.82 ± 0.10^Db^	−1.78 ± 0.21^Da^
b* value	A	−3.39 ± 0.03^Ad^	−3.28 ± 0.02^Ac^	−3.23 ± 0.05^Ab^	−3.02 ± 0.02^Aa^
	B	−3.63 ± 0.04^Bd^	−3.61 ± 0.06^Bc^	−3.59 ± 0.11^Bb^	−3.56 ± 0.04^Ea^
	C	−3.64 ± 0.02^Cd^	−3.61 ± 0.04^Bc^	−3.52 ± 0.08^Cb^	−3.46 ± 0.05^Ba^
	D	−3.66 ± 0.04^Dd^	−3.56 ± 0.06^Cc^	−3.51 ± 0.05^Db^	−3.47 ± 0.02^Ca^
	E	−3.75 ± 0.03^Ed^	−3.66 ± 0.05^Dc^	−3.60 ± 0.07^Eb^	−3.53 ± 0.02^Da^
	F	−3.78 ± 0.03^Fd^	−3.72 ± 0.06^Ec^	−3.68 ± 0.02^Fb^	−3.66 ± 0.04^Fa^

*Note*: The data are presented as mean ± standard deviation (*n* = 3). For every parameters, the data with different lowercase letters as superscript between rows (storage days) are significantly different (*p* < .05) and those with different uppercase letters as superscript between columns (treatments) are significantly different (*p* < .05). A (0‐mL soy whey, 100‐mL pineapple juice), B (10‐mL soy whey, 90‐mL pineapple juice), C (20‐mL soy whey, 80‐mL pineapple juice), D (30‐mL soy whey, 70‐mL pineapple juice), E (40‐mL soy whey, 60‐mL pineapple juice), and F (50‐mL soy whey, 50‐mL pineapple juice).

The a* value of the beverage samples decreased considerably (P < .05) from −1.68 to −1.89 with increase in the SW percentage. These results correlate with L* values as with increasing the soy whey percentage, L* values increasing thereby decreasing a* values. Similar diminish in the a* was also stated by Baba, Din, et al. (2016) and Baba, Rashid, et al. (2016) in milk‐whey‐treated fruit beverages. During storage, the a* increased significantly (*p* < .05), owing to the interaction between reducing sugars and amines (Gao et al., 2024). Deka et al. (2005) also identified a decrease in a* values of pineapple–mango spiced beverage samples during storage.

The b* values of the untreated/control beverage sample were higher (−3.02 to −3.39) than soy‐whey‐treated (−3.63 to −3.78) beverage samples. The higher b* of control may be attributed to the higher carotenoid content (Ali et al., 2020). With the incorporation of soy whey, there is more and more dilution due to the higher water content in soy whey. Punoo et al. (2023) also stated a higher b* value of control orange juice beverages compared to whey‐treated beverage formulations. Moreover, Vieira et al. (2020) also stated a decrease in b* values of orange juice‐based beverages treated with goat milk whey. During storage of 30 days, the b* values of both control and treated samples increased significantly owing to the Millard reaction as reported by Gao et al. (2024). Similar increase in b* values was also reported by Koffi et al. (2005) in UHT‐treated banana whey‐based beverage samples during storage.

#### 3.2.4 Rheological properties

The flow behavior and frequency sweep tests were conducted for evaluating the rheological properties of control and soy whey‐fortified beverage samples.

##### Flow behavior

**FIGURE 2:**
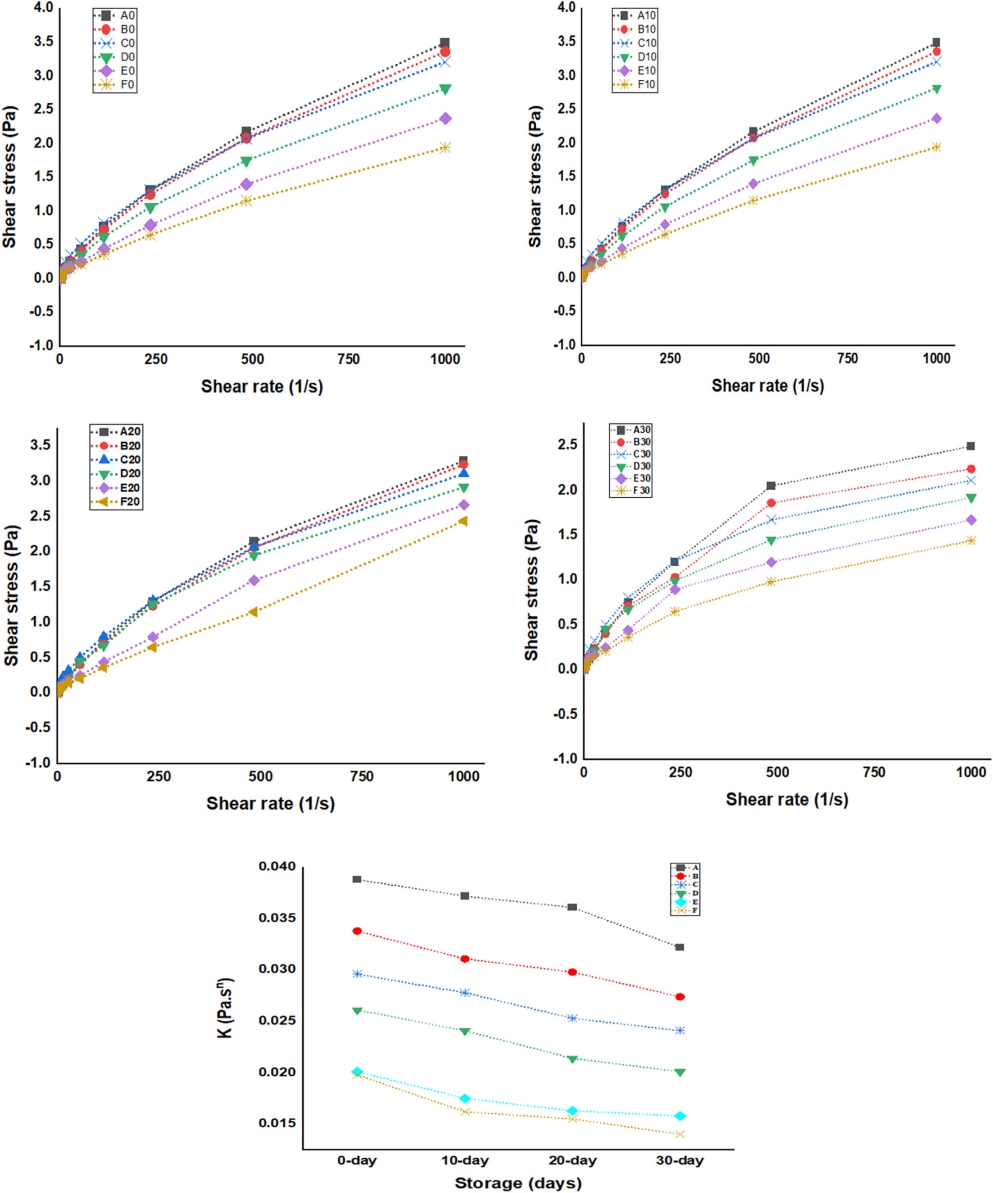
Flow behavior of soy whey‐fortified pineapple juice beverages, A (0‐mL soy whey, 100‐mL pineapple juice), B (10‐mL soy whey, 90‐mL pineapple juice), C (20‐mL soy whey, 80‐mL pineapple juice), D (30‐mL soy whey, 70‐mL pineapple juice), E (40‐mL soy whey, 60‐mL pineapple juice), and F (50‐mL soy whey, 50‐mL pineapple juice).

The shear stress (SS) and shear rate (SR) of beverage formulations were fixed into the power‐law equation to model the flow behavior as revealed in Figure 2. The parameters of equation such as consistency coefficient (*K*) and flow index (*n*) were explained using SS and SR and rate data to model relationship between the SS and SR of beverage formulations. The model fitness depends on the *R* ^2^ value, as *R* ^2^ is close to the one stated the best model fit. The *n* = 1, *n* < 1.0, and *n* > 1.0 depict Newtonian, shear‐thinning, and shear‐thickening behavior considerably (Silva et al., 2019). The n value of the formulations ranged between 0.4758 and 0.6521, with a yield stress of 0.018–0.025 Pa, hence the beverages depicted shear‐thinning behavior with Herschel–Bulkley property. Similar response was also stated by Vieira et al. (2020) for milk‐whey‐based orange beverages. Jokar and Azizi (2022) stated yield stress increment of beverages from 1.43 to 7.12 pa with an escalation of fruit juice content from 10% to 20%. Moreover, Silva et al. (2019) also identified Herschel–Bulkley character of phyto‐beverages with n value ranging between 0.710 and 0.825. Similarly, Lopes et al. (2019) reported this behavior in ultrahigh‐temperature‐treated nut beverages. The *K* value of the developed beverage formulations ranged between 0.0198 and 0.0388 Pa∙S^n^, and with increase in the whey percentage of formulations, the *K* decreased considerably (*p* < .05). Lopes et al. (2019) stated *K* values of 0.038, 0.358, and 0.047 Pa∙S^n^ for various nut‐based beverages. Additionally, the apparent viscosity in developed beverage formulations decreased with an escalation in the shear rate (s^‒1^). Meanwhile, viscosity is associated with intermolecular interactions, rise in the rotating speed changed the intermolecular attractions in the beverage formulations, resulting in variation in molecular characteristics of beverage formulations, and hence there is an alteration in their viscosity (Cui et al., 2022). As depicted in Figure 2, the consistency coefficient (*K*) of the beverage samples decreased significantly (*p* < .05). The results correlate with the TSS and pH of the present investigation as during storage pH decreased and TSS increased significantly (*p* < .05). The decrease in the consistency coefficient may be attributed to the hydrolysis of polymeric substances in the beverage samples as reported by Yadav et al. (2013). Dhiman et al. (2017) reported that with the increase during storage of beverage samples, the polysaccharides get converted to the simple sugars.

##### Frequency sweep

**FIGURE 3:**
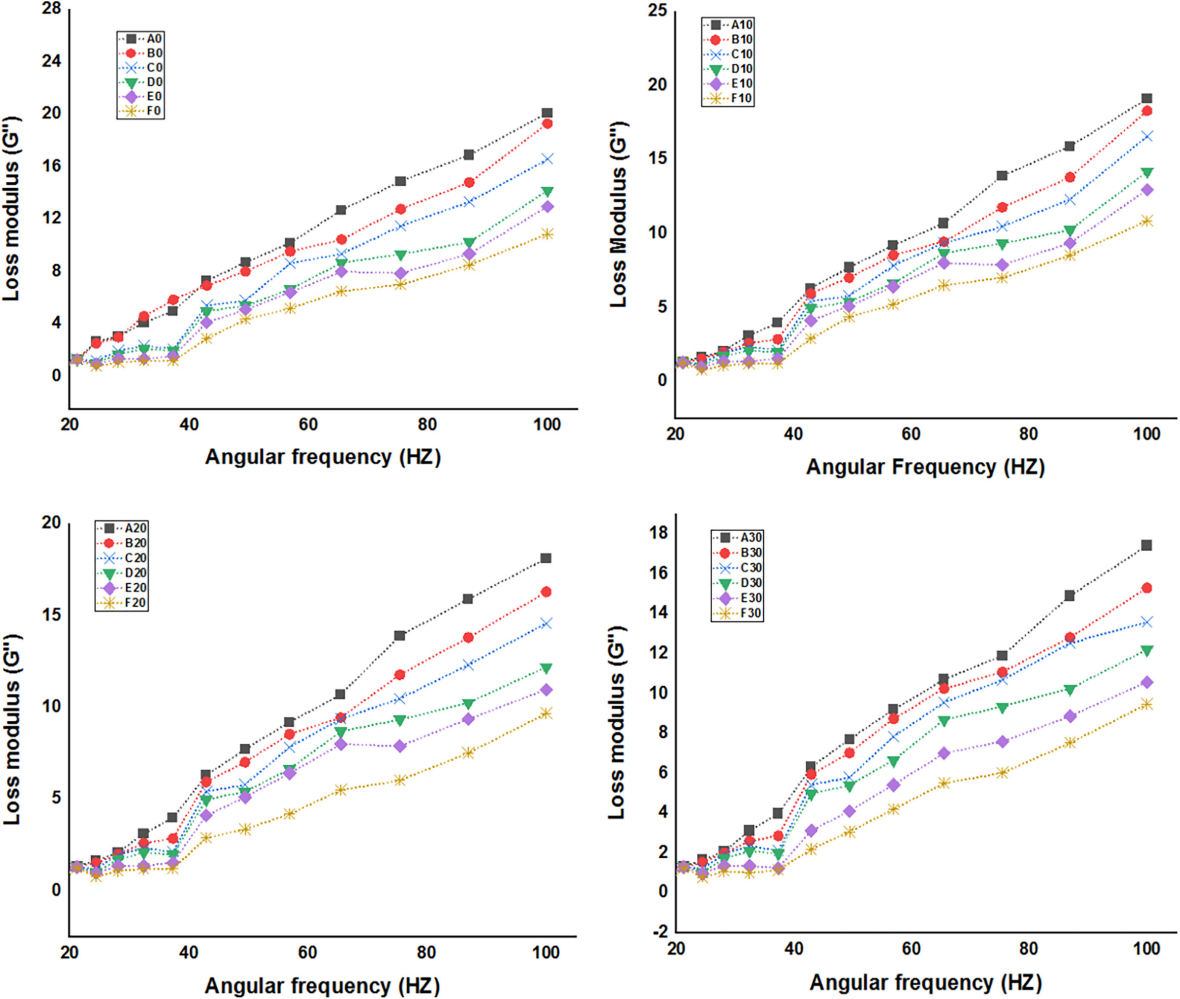
Loss Modulus (*G*″) of soy whey‐fortified pineapple juice beverages, A (0‐mL soy whey, 100‐mL pineapple juice), B (10‐mL soy whey, 90‐mL pineapple juice), C (20‐mL soy whey, 80‐mL pineapple juice), D (30‐mL soy whey, 70‐mL pineapple juice), E (40‐mL soy whey, 60‐mL pineapple juice), and F (50‐mL soy whey, 50‐mL pineapple juice).

**FIGURE 4:**
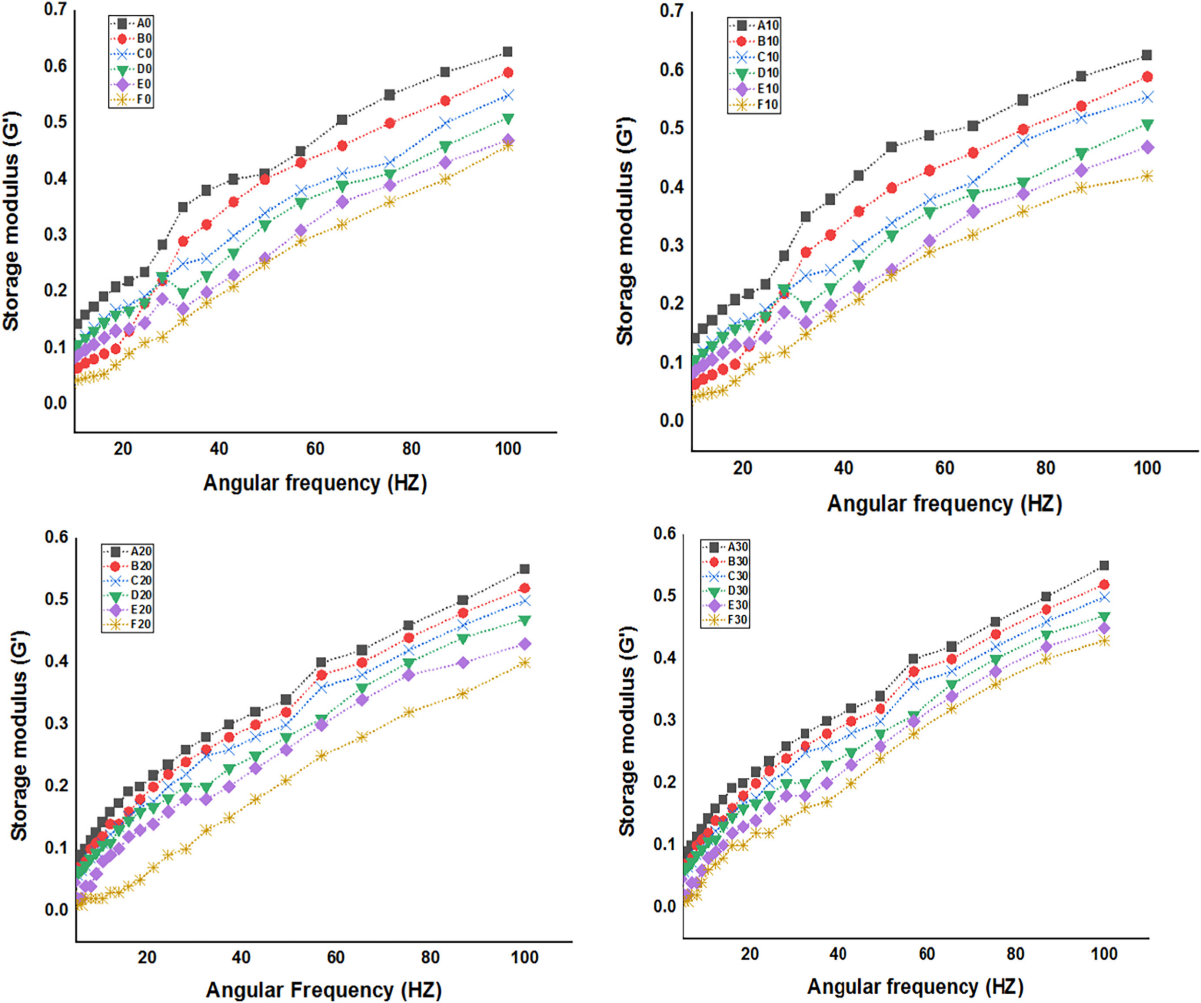
Storage modulus (*G*″) of soy whey‐fortified pineapple juice beverages, A (0‐mL soy whey, 100‐mL pineapple juice), B (10‐mL soy whey, 90‐mL pineapple juice), C (20‐mL soy whey, 80‐mL pineapple juice), D (30‐mL soy whey, 70‐mL pineapple juice), E (40‐mL soy whey, 60‐mL pineapple juice), and F (50‐mL soy whey, 50‐mL pineapple juice).

It is evident from Figures 3 and Figure 4 that the beverage samples' *G*″ values were higher than their *G′* values. This is because beverages tend to have a higher fluid character than solid character, as prime quality attribute of beverages. Increasing soy whey concentration in the developed beverages decreased *G*′ values significantly (*p* < .05). These results correlate with the TSS results as with increase in soy concentration, the TSS decreased significantly. As the frequency intensified, the sample *G*′ and *G*″ increased and showed weaker gel dynamic oscillatory characteristics. Cui et al. (2022) stated that the changes in *G*″ and *G*′ could be attributed to the elimination of chemical interactions between molecules caused by an increase in frequency. Changes in the chemical content and particle size of beverage formulations during the homogenization could be the cause of the variation in *G*′ and *G*″ in the samples (Tan & Kerr, 2015). Cui et al. (2022) also observed similar response in beverage samples made with chestnut lilies when they homogenized the samples at different rotating rates. During storage, the storage modulus as well as loss modulus values decreased considerably (*p* < .05). Decrease in *G*′ and *G*″ values correlates with pH results, diminish in pH during storage resulted in hydrolysis of polymeric substances of beverages, thereby reducing *G*′ and *G*″ (Baydin et al., 2022). Similar diminish in *G*′ and *G*″ values was also reported by Paquet et al. (2014) in fruit juice beverages during storage treated with dietary fiber and xanthan gum. Cho and Yoo (2015) also reported similar results in cold thickened beverages during storage treated with xanthan gum.

#### 3.2.5 Antioxidant activity

The DPPH (%), reducing power (%), and ABTS (%) inhibition activity of the SW‐fortified pineapple beverage are shown in the Table 3. With increment in percentage of soy whey from 0% to 30%, the DPPH (%) capacity of the beverage formulations increased considerably (*p* < .05) from 56.58% to 57.01%. The increase in DPPH (%) scavenging of beverage formulations may be attributed to the presence of phenolic substances such as daidzein, genistein, and glycitin in SW as stated by Kulprachakarn et al. (2021). The beverage samples with 40% and 50% SW concentrations showed the lowest DPPH (%) inhibition activity. Baba, Din, et al. (2016) and Baba, Rashid, et al. (2016) also reported a decrease in DPPH (%) activity of pineapple beverage samples incorporated with 10%–30% cheese and paneer whey. Additionally, Punoo et al. (2023) also stated escalation in DPPH (%) activity of orange juice beverage samples amalgamated up to 30% soy whey and samples with above 30% soy whey showed lower DPPH (%) activity. With increase in the storage period from 10 to 30 days, the DPPH (%) activity of both control and soy whey‐treated pineapple beverage samples decreased considerably (*p* < .05). Similar diminish in DPPH (%) inhibition activity was also stated by Rau De Almeida Callou et al. (2010) in soy‐based beverages during storage.

**TABLE 3:** Antioxidant activity of control and soy whey‐treated beverage samples during storage.

Parameter	Sample	Storage days			
		0th day	10th day	20th day	30th day
DPPH (%)	A	56.58 ± 0.05^Ca^	56.52 ± 0.12^Cb^	55.46 ± 0.05^Cc^	53.11 ± 0.11^Cd^
	B	56.79 ± 0.22^Ba^	56.77 ± 0.08^Bb^	56.12 ± 0.21^Ac^	55.23 ± 0.10^Ad^
	C	57.01 ± 0.20^Aa^	56.91 ± 0.03^Ab^	55.11 ± 0.09^Dc^	53.82 ± 0.04^Bd^
	D	56.32 ± 0.08^Da^	56.28 ± 0.14^Db^	55.72 ± 0.06^Bc^	51.52 ± 0.07^Dd^
	E	55.85 ± 0.04^Ea^	54.11 ± 0.07^Eb^	51.80 ± 0.12^Ec^	50.75 ± 0.22^Ed^
	F	55.42 ± 0.21^Fa^	53.27 ± 0.20^Fb^	50.48 ± 0.06^Fc^	50.01 ± 0.30^Fd^
Reducing power (%)	A	46.84 ± 0.20^Ca^	44.36 ± 0.15^Db^	43.52 ± 0.08^Dc^	41.20 ± 0.05^Dd^
	B	46.88 ± 0.02^Ba^	44.56 ± 0.05^Cb^	43.57 ± 0.03^Cc^	41.29 ± 0.10^Cd^
	C	46.96 ± 0.05^Ba^	44.71 ± 0.14^Bb^	43.69 ± 0.09^Bc^	41.54 ± 0.13^Bd^
	D	47.01 ± 0.21^Aa^	45.82 ± 0.10^Ab^	43.80 ± 0.04^Ac^	41.71 ± 0.06^Ad^
	E	45.28 ± 0.08^Da^	44.83 ± 0.05^Eb^	42.48 ± 0.02^Ec^	40.25 ± 0.12^Ed^
	F	45.04 ± 0.05^Ea^	43.12 ± 0.12^Fb^	41.09 ± 0.14^Fc^	40.11 ± 0.08^Fd^
ABTS (%)	A	56.35 ± 0.21^Ca^	55.41 ± 0.05^Db^	53.57 ± 0.02^Dc^	51.28 ± 0.10^Dd^
	B	56.54 ± 0.23^Ca^	55.45 ± 0.08^Cb^	54.31 ± 0.06^Cc^	52.54 ± 0.05^Cd^
	C	56.83 ± 0.05^Ba^	55.51 ± 0.04^Bb^	54.66 ± 0.05^Bc^	52.83 ± 0.02^Bd^
	D	56.90 ± 0.08^Aa^	55.92 ± 0.12^Ab^	55.11 ± 0.02^Ac^	53.90 ± 0.02^Ad^
	E	55.11 ± 0.13^Ea^	54.21 ± 0.09^Eb^	53.21 ± 0.10^Ec^	51.22 ± 0.11^Ed^
	F	54.87 ± 0.11^Fa^	53.37 ± 0.02^Fb^	51.73 ± 0.12^Fc^	50.87 ± 0.04^Fd^

*Note*: The data are presented as mean ± standard deviation (*n* = 3). For every parameters, the data with different lowercase letters as superscript between rows (storage days) are significantly different (*p* < .05) and those with different uppercase letters as superscript between columns (treatments) are significantly different (*p* < .05). A (0‐mL soy whey, 100‐mL pineapple juice), B (10‐mL soy whey, 90‐mL pineapple juice), C (20‐mL soy whey, 80‐mL pineapple juice), D (30‐mL soy whey, 70‐mL pineapple juice), E (40‐mL soy whey, 60‐mL pineapple juice), and F (50‐mL soy whey, 50‐mL pineapple juice).

Reducing power (%) activity of the control and soy whey‐fortified (0%–30%) beverage samples increased considerably from 46.84% to 47.01%. This increase in reducing power by incorporation of soy whey (0%–30%) may be owing to the chelating activity of phytochemicals such as daidzein, genistein, and glycitin of the soy whey (Manayi, 2021). From 40% to 50% soy whey incorporation, the reducing power gets decreased. Baba, Din, et al. (2016) also reported a decrease in reducing power activity of pineapple beverage samples with paneer and cheese whey added as adjuncts. With escalation in storage from 0 to 30 days, the reducing power activity (%) decreased considerably (*p* < .05). This decrease in reducing power during storage may be owing to the deprivation of phenolic substances during storage (Rocha‐Parra et al., 2016).

Similar to that of DPPH (%) inhibition and reducing power activity, the 0%, 10%, 20%, and 30% SW‐treated pineapple beverage formulations showed greater ABTS (%) activity than 40% and 50% SW‐treated beverage samples. Dai et al. (2021) stated that phenolic substances and flavonoids of soy whey disturb chain oxidative reactions as having strong hydrogen donation and metal chelation activity. Similar consequences were also stated by Punoo et al. (2023) in orange juice beverages treated with tofu whey. With rise in the storage period from 0 to 30 days, the ABTS activity of control and SW‐treated beverage samples decreased considerably (*p* < .05). During storage, there is a reduction in the pH of beverages as discussed in the pH of beverages during storage. This reduction in the pH during storage affects protonation and deprotonation of the phenolics of soy whey and pineapple juice which leads to decrease in the radical capturing activity of these phenolics (Li et al., 2020).

#### 3.2.6 Microbial analysis of beverages

The total plate count (TPC) values of the soy whey‐fortified pineapple beverage samples are shown in Table 4. The total plate count of control and soy whey‐treated beverage samples was below the permissible limit of 50 log CFU/mL throughout the storage of 30 days. On zero days, no colony of microbes was detected in the samples, which may be owing to the impact of sterilization during the processing of the beverage. From the 10th day up to 30th day, the colony count increases from 2.7 to 4.32 log CFU/mL for the control sample. The TPC results of the soy whey‐fortified pineapple beverage formulations are inconsistent with Gorachiya et al. (2018), Sharma et al. (2020), and Mohamed et al. (2014) who stated alike trend of significant escalation in the TPC of dairy whey beverage formulations with increasing storage. Additionally, Ahmed et al. (2023) also stated an increase in TPC of dairy whey‐based fruit beverages. With an increase in the percentage of SW in developed beverages, the TPC count decreased considerably (*p* < .05). This decrease in TPC count may be owing to the greater phenolic contents of SW as stated by Kulprachakarn et al. (2021), due to the presence of daidzein, genistein, and epigenistin content of soy whey.

**TABLE 4:** Microbiological analysis of beverage samples during storage.

Parameter	Sample TPC	0th day	10th day	20th day	30th day
TPC (log CFU/mL)	A	ND	2.775 ± 1.0^Ac^	3.898 ± 0.5^Ab^	4.321 ± 0.8^Aa^
	B	ND	2.443 ± 0.5^Bc^	3.881 ± 0.8^Bb^	4.235 ± 1.0^Ba^
	C	ND	2.278 ± 0.8^Cc^	3.857 ± 1.5^Cb^	4.124 ± 1.2^Ba^
	D	ND	1.740 ± 1.0^Dc^	3.785 ± 1.0^Cb^	4.021 ± 0.7^Ca^
	E	ND	1.685 ± 1.2^Ec^	2.748 ± 0.4^Db^	3.852 ± 0.4^Da^
	F	ND	1.653 ± 1.0^Fc^	2.716 ± 0.7^Eb^	3.435 ± 1.0^Ea^
Yeast and mold (log CFU/mL)	A	ND	ND	1.981 ± 0.08^Ab^	2.124 ± 0.08^Aa^
	B	ND	ND	1.972 ± 1.20^Bb^	1.991 ± 1.20^Ba^
	C	ND	ND	1.964 ± 1.02^Cb^	1.972 ± 1.02^Ca^
	D	ND	ND	1.625 ± 0.03^Db^	1.945 ± 0.03^Da^
	E	ND	ND	1.422 ± 0.05^Eb^	1.920 ± 0.05^Ea^
	F	ND	ND	1.231 ± 0.08^Fb^	1.921 ± 0.08^Fa^

*Note*: The data are presented as mean ± standard deviation (*n* = 3). For every parameters, the data with different lowercase letters as superscript between rows (storage days) are significantly different (*p* < .05) and those with different uppercase letters as superscript between columns (treatments) are significantly different (*p* < .05). A (0‐mL soy whey, 100‐mL pineapple juice), B (10‐mL soy whey, 90‐mL pineapple juice), C (20‐mL soy whey, 80‐mL pineapple juice), D (30‐mL soy whey, 70‐mL pineapple juice), E (40‐mL soy whey, 60‐mL pineapple juice), and F (50‐mL soy whey, 50‐mL pineapple juice).

Up to the 10th day of storage, neither the control nor the soy whey‐treated samples showed any signs of yeast or mold. On the 20th day, the control sample showed 1.981 log CFU/mL of yeast and mold count which increased to 2.124 log CFU/mL on the 30th day of storage. The yeast and mold count of control sample was above permissible limit of 2 log CFU/mL on 30th day of storage. Ahmed et al. (2023) stated TPC of 1.71, 1.78, and 1.85 log CFU/mL of yeast and mold in cheese whey‐fortified fruit beverages after 25 days of storage. Similar trending consequences of yeast and mold count were also perceived by Yonis et al. (2014) in guava–whey beverage samples, Gorachiya et al. (2020) in dairy whey beverage samples, and Sharma et al. (2021) in Aloe vera and coconut water‐based beverage samples. With increment in percentage of soy whey in developed soy whey‐fortified beverage, the yeast and mold count decreased significantly (*p* < .05), and the yeast and mold count was below permissible limit of 2.0 log CFU/mL. Similar results were also stated by Punoo et al. (2023) in orange beverages fortified with tofu whey. This decrease in TPC count may be attributed to the higher phenolic contents of soy whey as stated by Kulprachakarn et al. (2021), owing to the presence of daidzein, genistein, and epigenistin content of soy whey.

#### 3.2.7 Sensory evaluation

The sensory scores for color, flavor, taste, consistency, and overall acceptability of the beverage formulations are shown in Table 5. With an increment in the soy whey percentage from 10% to 30%, the color, flavor, taste, and overall acceptability increased significantly (*p* < .05) from 8.0–8.5, 7.9–8.4, 8.0–8.4, and 7.9–8.3, respectively. The beverage formulations with 40% and 50% soy whey showed lower sensory scores, whereas the beverage sample incorporated with 30% soy whey showed higher color, flavor, consistency, taste, and overall acceptability results. Comparable results were also stated by Punoo et al. (2023) in orange juice beverages fortified with soy whey. Baba, Din, et al. (2016) and Baba, Rashid, et al. (2016) also reported a decrease in sensory attributes of pineapple beverage incorporated with 10% cheese whey and below 10% cheese whey incorporation showed enhanced sensory properties. This decrease in sensory scores of 40% and 50% soy whey‐containing formulations may be attributed to the higher dilution factor (Punoo et al., 2023). Additionally, Zhao et al. (2021) stated that the decrease in the sensory score of food product with increase in soy‐based ingredient may be owing to the increase in beany flavor of the product. With the increase in the storage time (days), the sensory score of both control and soy‐treated beverage samples decreased significantly. This decrease in the sensory parameter score may be owing to the increase in auto‐oxidative and chemical degradation of beverage components as reported by Mu et al. (2022).

**TABLE 5:** Sensory analysis of beverage samples during storage.

Storage days	Sample ID	Color	Flavor	Taste	Consistency	Overall acceptability
0th Day	A	8.0 ± 0.03^D^	7.9 ± 0.02^D^	8.0 ± 0.05^D^	7.8 ± 0.04^A^	7.9 ± 0.05^D^
	B	8.1 ± 0.04^C^	8.0 ± 0.04^C^	8.2 ± 0.03^C^	7.7 ± 0.05^B^	8.0 ± 0.02^C^
	C	8.2 ± 0.02^B^	8.2 ± 0.03^B^	8.2 ± 0.02^B^	7.6 ± 0.03^C^	8.1 ± 0.04^B^
	D	8.5 ± 0.02^A^	8.4 ± 0.03^A^	8.4 ± 0.01^A^	7.6 ± 0.03^C^	8.3 ± 0.06^A^
	E	7.6 ± 0.03^E^	7.7 ± 0.04^E^	7.7 ± 0.03^E^	7.0 ± 0.02^D^	7.2 ± 0.02^E^
	F	7.3 ± 0.05^F^	7.1 ± 0.02^F^	7.2 ± 0.05^F^	7.0 ± 0.04^D^	7.0 ± 0.04^F^
10th Day	A	8.0 ± 0.03^C^	7.7 ± 0.03^D^	7.8 ± 0.05^C^	7.6 ± 0.04^C^	7.6 ± 0.02^D^
	B	8.0 ± 0.05^C^	8.0 ± 0.05^C^	7.9 ± 0.02^B^	7.7 ± 0.03^B^	7.9 ± 0.04^C^
	C	8.1 ± 0.01^B^	8.1 ± 0.04^B^	7.9 ± 0.04^B^	7.8 ± 0.02^A^	8.0 ± 0.02^B^
	D	8.3 ± 0.04^A^	8.2 ± 0.02^A^	8.0 ± 0.02^A^	7.8 ± 0.02^A^	8.1 ± 0.02^A^
	E	7.4 ± 0.03^D^	7.5 ± 0.02^E^	7.2 ± 0.03^D^	6.9 ± 0.04^D^	7.0 ± 0.02^E^
	F	7.0 ± 0.04^E^	7.0 ± 0.03^F^	7.0 ± 0.03^E^	6.7 ± 0.05^E^	6.9 ± 0.05^F^
20th Day	A	7.8 ± 0.03^C^	7.5 ± 0.05^C^	7.6 ± 0.01^C^	7.4 ± 0.07^C^	7.5 ± 0.05^C^
	B	7.8 ± 0.04^C^	7.9 ± 0.05^B^	7.7 ± 0.03^B^	7.5 ± 0.05^B^	7.7 ± 0.04^B^
	C	7.9 ± 0.02^B^	7.9 ± 0.02^B^	7.7 ± 0.02^B^	7.7 ± 0.01^A^	8.0 ± 0.05^A^
	D	8.0 ± 0.02^A^	8.0 ± 0.03^A^	8.0 ± 0.03^A^	7.7 ± 0.04^A^	8.0 ± 0.03^A^
	E	7.2 ± 0.03^D^	7.2 ± 0.02^D^	6.9 ± 0.03^D^	6.5 ± 0.04^D^	6.9 ± 0.03^D^
	F	6.9 ± 0.05^E^	7.0 ± 0.03^E^	6.8 ± 0.04^E^	6.3 ± 0.03^E^	6.7 ± 0.03^E^
30th Day	A	7.6 ± 0.06^C^	7.5 ± 0.05^D^	7.5 ± 0.05^C^	7.2 ± 0.07^C^	7.4 ± 0.04^D^
	B	7.7 ± 0.04^B^	7.7 ± 0.05^C^	7.5 ± 0.04^C^	7.4 ± 0.04^B^	7.6 ± 0.0^C^
	C	7.7 ± 0.03^B^	7.8 ± 0.02^B^	7.7 ± 0.04^B^	7.6 ± 0.06^A^	7.8 ± 0.02^B^
	D	8.0 ± 0.03^A^	7.9 ± 0.03^A^	7.9 ± 0.04^A^	7.6 ± 0.02^A^	7.9 ± 0.03^A^
	E	7.0 ± 0.05^D^	6.9 ± 0.03^E^	6.8 ± 0.02^D^	6.2 ± 0.04^D^	6.5 ± 0.01^E^
	F	6.7 ± 0.03^E^	6.8 ± 0.05^F^	6.8 ± 0.02^D^	6.2 ± 0.03^D^	6.5 ± 0.04^E^

*Note*: The data are presented as mean ± standard deviation (*n* = 3). For every parameters, the data with different lowercase letters as superscript between rows (storage days) are significantly different (*p* < .05) and those with different uppercase letters as superscript between columns (treatments) are significantly different (*p* < .05). A (0‐mL soy whey, 100‐mL pineapple juice), B (10‐mL soy whey, 90‐mL pineapple juice), C (20‐mL soy whey, 80‐mL pineapple juice), D (30‐mL soy whey, 70‐mL pineapple juice), E (40‐mL soy whey, 60‐mL pineapple juice), and F (50‐mL soy whey, 50‐mL pineapple juice).

## 4 CONCLUSION

Fortified drinks are gaining the attention of consumers owing to their nutritional profile and nutraceutical properties. Soy whey a byproduct of tofu product preparation is a rich source of phytochemicals and nutrients. Incorporation of soy whey up to 30% enhanced the nutraceutical potential of developed pineapple beverages significantly (*p* < .05) by enhancing DPPH, reducing power, and ABTS activity. Color and TSS of the beverages at these concentrations enhanced the quality attributes of developed beverages. The rheological quality attributes were improved by soy whey incorporation. Microbial evaluation indicated that increased soy whey concentration inhibited microbial growth significantly. In sensory evaluation, beverage samples up to 30% soy whey concentration improved the sensory attributes of beverages. Above 30% soy concentration decreased the overall quality attributes. Hence, up to 30% soy whey concentration, the nutraceutical as well as overall quality and consumer acceptability of beverages gets enhanced significantly. Further investigations are required to evaluate the impact of varying temperatures and relative humidity on the quality and shelf life of these fortified beverages.

## CONFLICT OF INTEREST STATEMENT

The authors declare that they have no known competing financial interests or personal relationships that could have appeared to influence the work reported in this paper.

## Data Availability

The data that support the findings of this study are available on request from the corresponding author.
